# Radiation-Induced Xerostomia in Patients With Head and Neck Cancer Treated With Comprehensive Salivary Gland-Sparing Helical Tomotherapy Technique Based on a Back Propagation Artificial Neural Network

**DOI:** 10.14740/jocmr6633

**Published:** 2026-09-26

**Authors:** Feng Teng, Qi Teng Liu, Ling Ling Meng, Zhong Jian Ju, Xiang Kun Dai, Xin Xin Zhang, Lin Ma

**Affiliations:** a Department of Radiotherapy, Beijing Tongren Hospital, Capital Medical University, Beijing 100730, China; b Department of Radiotherapy, Beijing Luhe Hospital, Affiliated to Capital Medical University, Beijing 101149, China; c Department of Radiation Oncology, First Medical Center of Chinese PLA General Hospital, Beijing 100853, China; d Department of Otorhinolaryngology Head and Neck Surgery, First Medical Center of Chinese PLA General Hospital, Beijing 100853, China; e Medical School of the Chinese PLA, Beijing 100853, China

**Keywords:** Head and neck cancer, Helical tomotherapy, Radiation-induced xerostomia, Prediction model, Back propagation artificial neural network (BPANN)

## Abstract

**Background:**

The aim was to create a back propagation artificial neural network (BPANN) model for gauging the risk of developing xerostomia (dry mouth) due to targeted radiotherapy in patients with head and neck cancer (HNC), who underwent comprehensive salivary gland-sparing helical tomotherapy (HT).

**Methods:**

Data from 222 HNC patients treated with salivary gland-sparing HT between November 26, 2016, and December 31, 2017, were analyzed. Potential variables considered included age, gender, tumor type, radiation dose to salivary glands, and xerostomia questionnaire scores. These variables were adjusted using multivariate linear regression. The BPANN model was constructed to predict the likelihood and severity of xerostomia at both 1 and 2 years after radiotherapy. Model evaluation was based on the confusion matrix table and the area under the receiver operating characteristic curve (AUC of ROC).

**Results:**

The BPANN model revealed that the risk of radiation-induced xerostomia could be evaluated using age, gender, tumor type, and radiation dose to specific salivary glands (parotid glands, submandibular glands, oral cavity, and tongue glands). Multivariate analysis indicated that age, gender, and submandibular gland dose were the primary influencing factors for xerostomia. Both prediction models demonstrated strong performance as reflected in the confusion matrix table and the AUC of ROC curve.

**Conclusions:**

BPANN stands as a potential and recommended predictive tool for assessing the likelihood of xerostomia induced by salivary gland-sparing HT.

## Introduction

Radiation-induced xerostomia is one of the most common and persistent toxicities in head and neck cancer (HNC) radiotherapy [1]. It markedly reduces quality of life by impairing swallowing, chewing, and taste, and increases the risks of dental caries and oral infections [2]. Current treatments such as oral hygiene care, artificial saliva, and salivary stimulants only offer symptomatic relief, with limited reversibility of glandular dysfunction [3]. Therefore, accurately predicting the risk and severity of xerostomia during radiotherapy planning is crucial for balancing tumor control and salivary gland protection [4].

The pathogenesis of xerostomia mainly results from radiation-induced salivary gland injury. With the development of intensity-modulated radiotherapy (IMRT), especially helical tomotherapy (HT), radiation dose to major salivary glands—including the parotid gland (PG), submandibular gland (SMG), tubarial gland (TG), and accessory salivary glands in the oral cavity (OC)—can be significantly reduced without compromising locoregional control [5]. However, despite these advances, xerostomia remains a major late complication among HNC survivors.

Traditional predictive models based on the Normal Tissue Complication Probability (NTCP) framework and logistic or LASSO regression primarily rely on linear assumptions and limited dose parameters, often focusing only on PG or SMG mean dose [6]. In reality, xerostomia is a multifactorial and nonlinear process influenced by glandular dose, age, sex, and tumor site [7]. Recent studies suggest that the newly identified TGs also contribute to baseline salivary secretion, warranting their inclusion in predictive modeling [8].

The back propagation artificial neural network (BPANN) is a machine-learning method capable of capturing nonlinear and interactive relationships without assuming variable independence [9]. Through iterative optimization, BPANN can better map complex associations between clinical and dosimetric inputs and xerostomia grades. Therefore, this study developed and validated a BPANN-based predictive model to estimate the 1-year and 2-year risk of xerostomia in patients treated with comprehensive salivary gland-sparing HT, integrating TG dose parameters for the first time in this context.

## Materials and Methods

### Participants and data collection

This was a retrospective analysis of prospectively collected data. A total of 246 inpatients with HNC who underwent comprehensive salivary gland-sparing HT were enrolled from the Department of Radiation Oncology at the First Medical Center of the Chinese People’s Liberation Army (PLA) General Hospital between November 26, 2016, and December 31, 2017. Twenty-four patients passed away or were lost to follow-up within 2 years after radiotherapy. The clinical characteristics of the remaining 222 patients are detailed in Table 1. Xerostomia risk factors, including age, gender, tumor type, PG right-side dose (PG-R), PG left-side dose (PG-L), SMG right-side dose (SMG-R), SMG left-side dose (SMG-L), OC dose, TG right-side dose (TG-R), TG left-side dose (TG-L), and xerostomia questionnaire (XQ) scores [10], were collected from each patient. This study was prospectively registered in the Chinese Clinical Trial Registry (ChiCTR-ONN-17010597) and approved by the Institutional Review Board of the First Medical Center, Chinese PLA General Hospital (Institutional Review Board (IRB) No. S2016-122-01). Written informed consent was obtained from all adult participants prior to enrollment. For participants under the legal age of consent, written informed consent from a parent or legal guardian was obtained, and age-appropriate assent was sought from the minors. No consent procedures were waived. All research procedures adhered to relevant institutional and international guidelines and regulations, as well as the Declaration of Helsinki.

**Table 1:** Patient Characteristics (N = 222)

Characteristics	Number of patients (%)
Age (years)	
Mean	49
Median (range)	52 (10–83)
Gender	
Male	164 (73.87)
Female	58 (26.13)
Tumor type	
Nasopharyngeal cancer	181 (81.53)
Oral cavity and oropharyngeal cancer	12 (5.41)
Hypopharyngeal and laryngeal cancer	19 (8.56)
Nasal cavity and paranasal sinuses cancer	10 (4.50)
PG-R/L (Gy)	
Mean	29.86/29.75
Median (ranged)	29.52/29.59 (19.45–62.86/18.28–47.79)
SMG-R/L (Gy)	
Mean	42.78/45.04
Median (range)	33.49/39.97 (21.37–71.63/22.38–69.56)
OC (Gy)	
Mean	32.39
Median (range)	31.91 (19.15–54.10)
TG-R/L (Gy)	
Mean	62.84/63.18
Median (range)	67.54/67.64 (9.46–71.73/9.41–71.74)
XQ score, 1 year	
Mean	8.74
Median (range)	8.00 (0–23)
No xerostomia	50 (22.52)
Mild xerostomia	105 (47.30)
Moderate/severe xerostomia	67 (30.18)
XQ score, 2 years	
Mean	4.44
Median (range)	3.00 (0–24)
No xerostomia	155 (69.82)
Mild xerostomia	46 (20.72)
Moderate/severe xerostomia	21 (9.46)

XQ score categories: 0–5, no xerostomia; 6–10, mild xerostomia; 11–20, moderate xerostomia; and 21–30, severe xerostomia. XQ: xerostomia questionnaire; PG: parotid gland; SMG: submandibular gland; OC: accessory salivary glands in the oral cavity; TG: tubarial gland; R: right; L: left.

### Treatment and xerostomia evaluation

Patients underwent comprehensive salivary gland-sparing HT. Prescription doses to irradiation areas ranged from 54 to 70 Gy over 30 to 33 fractions. Efforts were made to minimize mean doses to relevant salivary glands, including PGs, SMGs, TGs, and OC. Target delineation adhered to the International Commission on Radiation Units and Measurements (ICRU) report 83 guidelines, supplemented by department-specific criteria for salivary gland protection [10]. Target volumes were depicted as illustrated in Figure 1. Treatment was administered using a TomoTherapy System (Accuray, USA). Xerostomia evaluation was performed using questionnaires at 1 and 2 years after radiotherapy, as previously validated and described [10].

**Figure 1:**
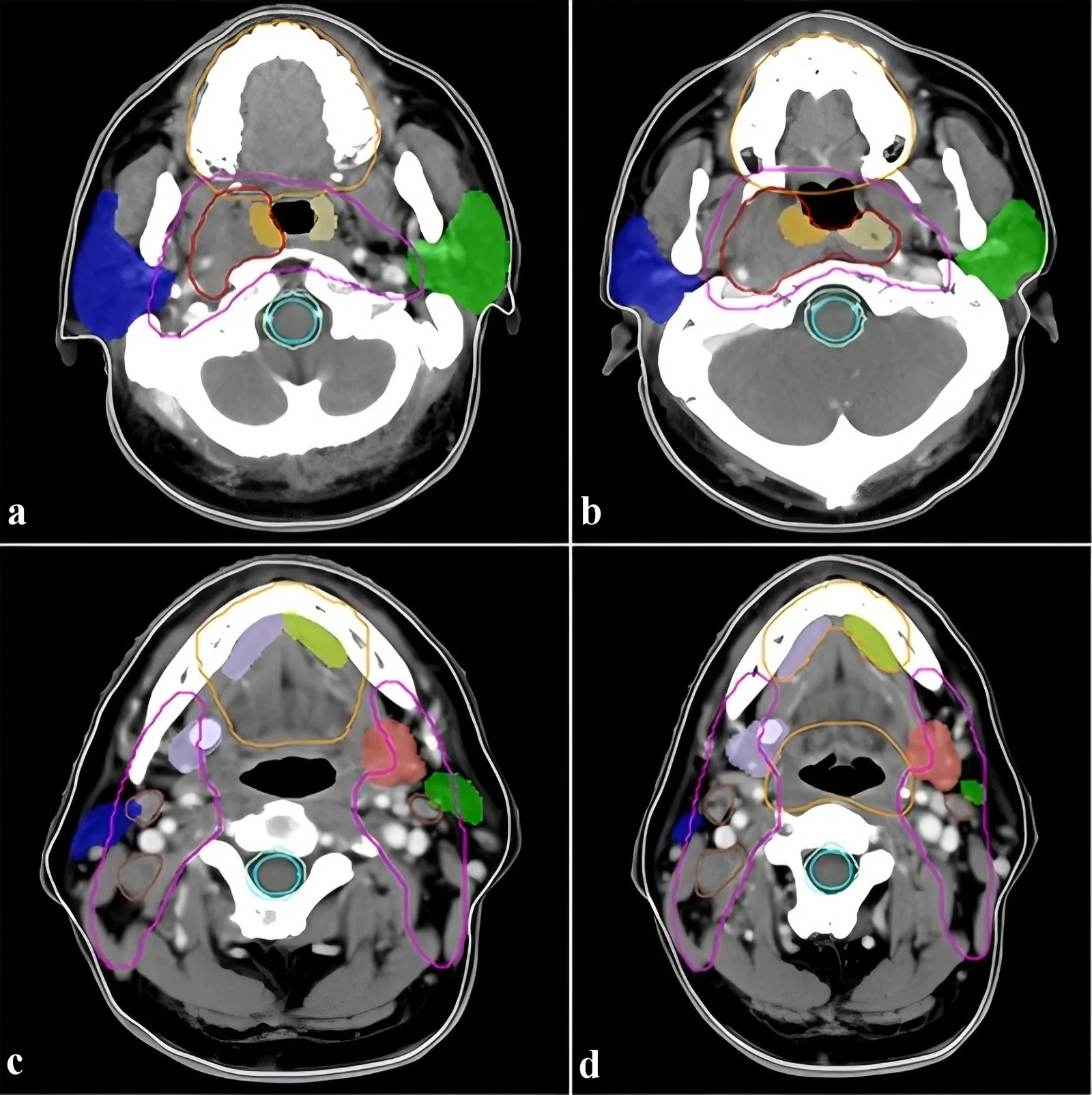
Illustration of target volume and salivary glands (fixed four layers). Panels a–d display different anatomical structures of the head and neck across four fixed layers, with each panel presenting a different view of the tumor volume, target volumes, and key salivary glands. Red line: GTVnx; brown line: GTVnd; pink line: CTV1; orange line: oral cavity; blue and green blocks: parotid glands; bright red and bright blue blocks: submandibular glands; yellow and faint yellow blocks: tubarial glands. GTVnx: gross tumor volume of nasopharynx; GTVnd: gross tumor volume of lymph nodal; CTV1: clinical target volume 1.

### Statistical analyses

Baseline characteristics such as age, gender, tumor type, radiation doses to salivary glands (PG-R, PG-L, SMG-R, SMG-L, OC, TG-R, TG-L), and XQ scores were presented in Table 1. Continuous variables were expressed as mean (standard deviation) or median (interquartile range), while categorical variables were presented as counts (percentages). XQ scores were categorized into: 0–5 (no xerostomia), 6–10 (mild xerostomia), 11–20 (moderate xerostomia), and 21–30 (severe xerostomia). Follow-up periods were at the 1-year and 2-year marks after radiotherapy. Spearman’s rank correlation test was used to determine the variables to be incorporated into the BPANN model. Model stability was assessed using intraclass correlation coefficients (ICCs) calculated from these measures obtained through cross-validation folds. All statistical analyses were performed using R (version 4.0.2) software, with a two-sided P value < 0.05 considered statistically significant.

### The BPANN prediction model

All participants (n = 222) were randomly divided into two sets: a training dataset (n = 159) and a test dataset (n = 63), maintaining a 7:3 ratio. The development and refinement of models were accomplished through these distinct datasets. Machine learning algorithms naturally fine-tune their feature parameters by autonomously capturing the connection between input and output data. Artificial neural networks (ANNs), an extension of logistic regression with added hidden layers, serve as common tools for training ANNs, particularly for complex deep neural networks. The process of backpropagation, involving iterative training, substantially enhances the model’s construction stability.

The training dataset was utilized to educate the model. Here, the XQ scores at 1 year and 2 years served as dependent variables. Independent variables encompassed age, gender, tumor type, and means of PG-R, PG-L, SMG-R, SMG-L, OC, TG-R, and TG-L doses. All independent variables underwent standardization before being included in the model. The ANN model was established by introducing the independent and dependent variables. The ggplot function was employed for the calculation and visualization of connection weights. The test dataset assessed the predictive capability of the trained BPANN model. During validation, metrics like the receiver operating characteristic (ROC) curve, important variable area under the curve (AUC) values, and the confusion matrix table were computed.

## Results

### The baseline data of characteristics of all participants

A total of 222 patients were included in the study (Table 1). The majority were male (73.87%), with a median age of 52 years (range: 10–83 years). Among them, 181 had nasopharyngeal cancer (81.53%), 12 had OC and oropharyngeal cancer (5.41%), 19 had hypopharyngeal and laryngeal cancer (8.59%), and 10 had nasal cavity and paranasal sinuses cancer (4.50%). HT was used to minimize doses to PG-R, PG-L, SMG-R, SMG-L, OC, TG-R, and TG-L. Regarding xerostomia severity, these three severity subgroups formed the grouping basis for the Spearman correlation analysis in Table 2 and the classification labels for BPANN model prediction in Table 3. Fifty patients had no xerostomia (22.52%), 105 had mild xerostomia (47.30%), and 67 had moderate/severe xerostomia (30.18%) at 1 year after radiotherapy. Corresponding numbers at 2 years were 155 (69.82%), 46 (20.72%), and 21 (9.46%), respectively. The median follow-up time was 46 months (range: 28–56 months).

**Table 2:** Spearman’s Rank Correlation Test for Association Between XQ Score and Relevant Characteristics

Variables	XQ score (1 year)		XQ score (2 years)	
	R	P value	R	P value
Gender	0.098	0.144	0.154	0.022
Tumor type	−0.070	0.302	−0.048	0.475
Age	0.180	0.007	0.241	< 0.001
PG-R Dmean	−0.050	0.459	−0.073	0.279
PG-L Dmean	0.063	0.348	−0.010	0.882
SMG-R Dmean	0.093	0.166	0.128	0.037
SMG-L Dmean	0.120	0.075	0.153	0.022
OC Dmean	0.020	0.762	−0.005	0.941
TG-R Dmean	0.021	0.757	−0.052	0.444
TG-L Dmean	0.094	0.163	−0.011	0.873

XQ score categories: 0–5, no xerostomia; 6–10, mild xerostomia; 11–20, moderate xerostomia; 21–30, severe xerostomia. Supplementary subgroup sizes for correlation analysis (full cohort, n = 222): 1-year: no xerostomia = 50, mild = 105, moderate/severe = 67; 2-year: no xerostomia = 155, mild = 46, moderate/severe = 21. XQ: xerostomia questionnaire; PG: parotid gland; SMG: submandibular gland; OC: accessory salivary glands in the oral cavity; TG: tubarial gland; R: right; L: left; Dmean: mean dose.

**Table 3:** BPANN Model Predictions for Xerostomia at Different Follow-up Times Post-Radiotherapy

Predicted value	True value							
	No xerostomia		Mild xerostomia		Moderate/severe xerostomia		Sum	
	1 year	2 years	1 year	2 years	1 year	2 years	1 year	2 years
No xerostomia	9	45	1	1	2	2	12	48
Mild xerostomia	3	0	30	10	4	1	37	11
Moderate/severe xerostomia	1	0	3	0	10	4	14	4
Total	13	45	34	11	16	7	63	63

The total test set sample size is 63; true subgroup counts: 1-year true group: no xerostomia = 13, mild = 34, moderate/severe = 16; 2-year true group: no xerostomia = 45, mild = 11, moderate/severe = 7. BPANN: back propagation artificial neural network.

### Correlation analysis

Table 2 summarized factors correlating with patient-reported XQ scores based on the full cohort of 222 patients, with complete coverage of the three xerostomia severity subgroups: 50 patients with no xerostomia, 105 with mild xerostomia, 67 with moderate/severe xerostomia at 1 year; 155 with no xerostomia, 46 with mild xerostomia, 21 with moderate/severe xerostomia at 2 years. At 1 year post-radiotherapy, only age exhibited a significant correlation with XQ scores. However, at 2 years post-radiotherapy, age, gender, SMG-R mean dose (Dmean), and SMG-L Dmean were all correlated with XQ scores. This highlights the significant relationship between xerostomia severity and age, gender, and radiation dose to SMG.

### BPANN model construction and validation via the data in test set

An ANN model was constructed with 10 input variables: gender, tumor type, age, PG-R Dmean, PG-L Dmean, SMG-R Dmean, SMG-L Dmean, OC Dmean, TG-R Dmean, and TG-L Dmean. The output layer comprised the three grades of xerostomia (no xerostomia, mild and moderately/severe xerostomia). The BPANN models were trained through 85,429 and 187,816 steps for the 1-year and 2-year predictions, respectively (Fig. 2a, b). Training concluded when the absolute partial derivative of the error function dropped below 0.01, followed by error likelihood calculation using the Akaike information criterion (AIC).

**Figure 2:**
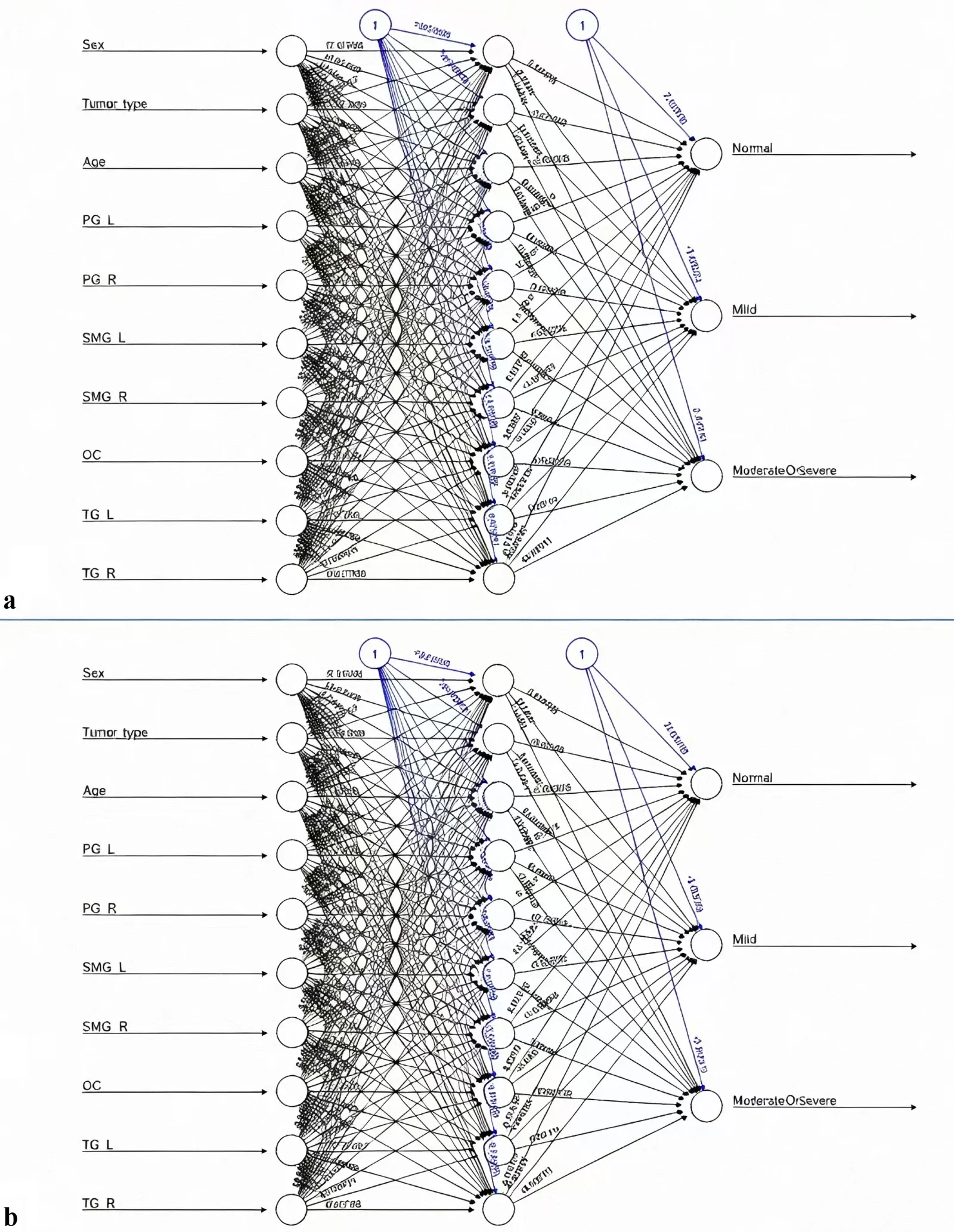
Topology diagrams of the BPANN prediction models at (a) 1 year and (b) 2 years post-radiotherapy. The input layer consists of the selected predictors including age, gender, tumor type, and bilateral radiation doses of parotid, submandibular, oral cavity and tubarial glands; the middle hidden layers represent the multilayer neural network architecture capturing nonlinear interactions among variables; the output layer generates three categories of predicted xerostomia risk (no xerostomia, mild xerostomia, moderate/severe xerostomia).Model training metrics are listed as follows: (a) 1-year prediction model: error = 0.37836, training steps = 85,429; (b) 2-year prediction model: error = 0.175809, training steps = 187,816. BPANN: back propagation artificial neural network.

The test set was employed to establish the predictive model. Table 3 lists the true case number of each xerostomia subgroup within this test set: at 1 year post-radiotherapy, there were 13 patients with no xerostomia, 34 with mild xerostomia, and 16 with moderate/severe xerostomia; at 2 years post-radiotherapy, there were 45 patients with no xerostomia, 11 with mild xerostomia, and seven patients with moderate/severe xerostomia. The projected probabilities of experiencing no xerostomia, mild xerostomia, and moderate/severe xerostomia 1 year after radiotherapy were 0.7500, 0.8108, and 0.7143, respectively (Table 3). Likewise, at the 2-year time point following radiotherapy, the prediction accuracies for no xerostomia, mild xerostomia, and moderate/severe xerostomia were 0.9375, 0.9091, and 1.000, respectively. The efficacy of the BPANN model was corroborated using ROC curve analysis. At the 1-year post-radiotherapy juncture, the areas under the ROC curve for no xerostomia, mild xerostomia, and moderate/severe xerostomia were 0.8835, 0.9474, and 0.9650, respectively. Similarly, at the 2-year post-radiotherapy interval, the areas under the ROC curve for no xerostomia, mild xerostomia, and moderate/severe xerostomia were 0.9417, 0.9000, and 0.8901, respectively (Fig. 3a, b). Predictive indicators of the BPANN model were evaluated, demonstrating that the predictive accuracy was 0.7778 (with a confidence interval of 0.6554 to 0.8728) at 1 year post-radiotherapy and 0.9365 (with a confidence interval of 0.8453 to 0.9824) at 2 years post-radiotherapy (Supplementary Materials 1, 2, jocmr.elmerjournals.com).

**Figure 3:**
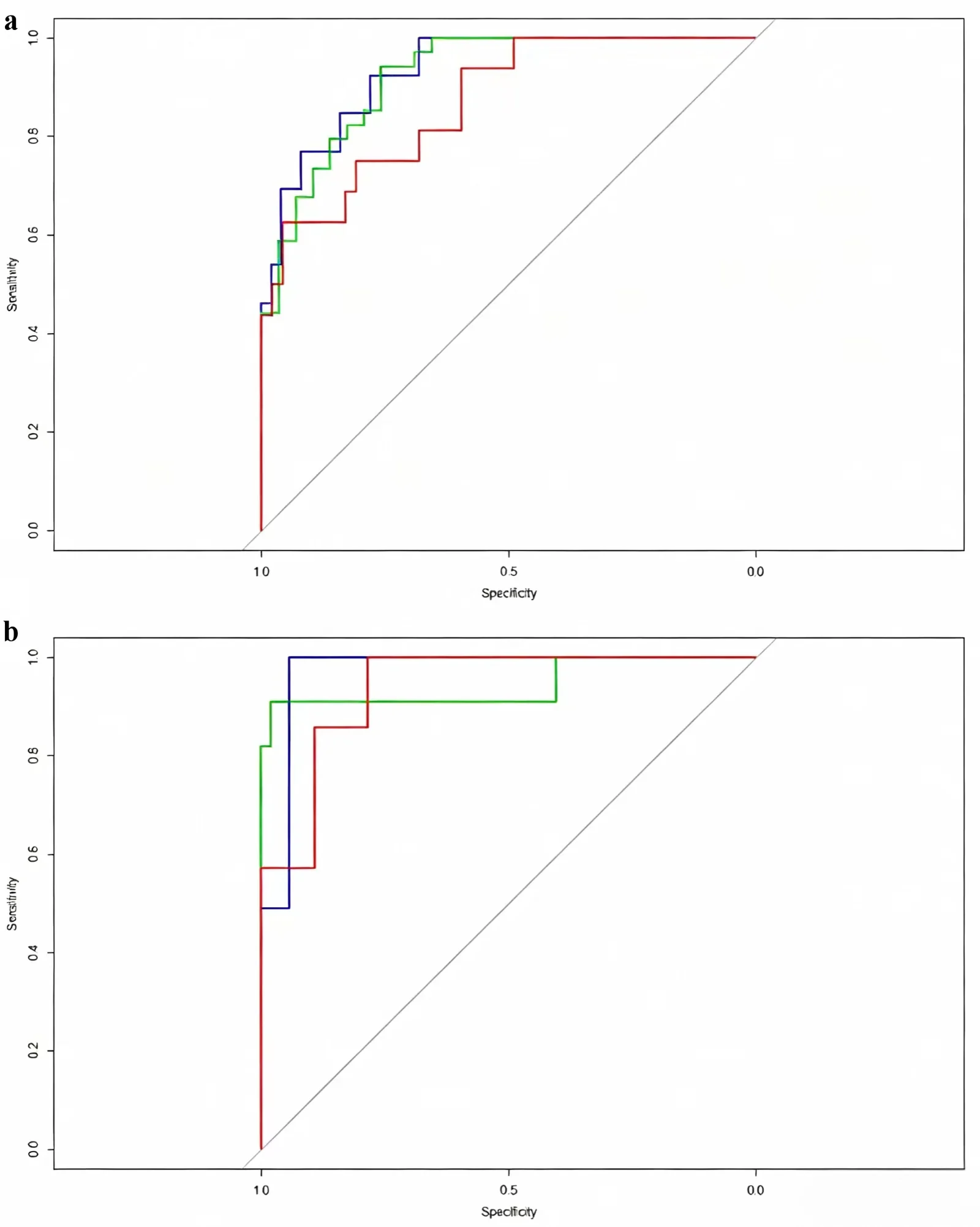
ROC curves of the BPANN prediction models for xerostomia risk at (a) 1 year and (b) 2 years post-radiotherapy. Blue curves represent the discrimination performance for patients without xerostomia, green curves for mild xerostomia, and red curves for moderate/severe xerostomia, with corresponding AUC values marked on each curve. AUC results: (a) 1-year model: no xerostomia = 0.8835, mild xerostomia = 0.9474, moderate/severe xerostomia = 0.9650; (b) 2-year model: no xerostomia = 0.9417, mild xerostomia = 0.9000, moderate/severe xerostomia = 0.8901. BPANN: back propagation artificial neural network; ROC: receiver operating characteristic.

## Discussion

Radiation-induced xerostomia remains one of the most prevalent and functionally disabling late toxicities following radiotherapy for HNC [1]. This symptom severely impairs swallowing, chewing, speech, and nutrition, thereby reducing long-term quality of life [11, 12]. Given its typically irreversible nature once established, early identification of high-risk patients and proactive salivary gland sparing are of paramount importance [13].

Traditional dose–response models, including the NTCP framework and multivariate logistic regression, have advanced understanding of xerostomia risk but often assume linearity and variable independence, which may oversimplify the complex biology of salivary gland injury [6, 14]. In recent years, machine-learning algorithms have shown great potential for modelling nonlinear, high-dimensional relationships [15, 16]. Among these, BPANNs offer an effective tool for toxicity prediction by automatically learning patient-specific correlations between clinical, anatomical, and dosimetric variables [9].

The BPANN algorithm, grounded in the multilayer perceptron (MLP) architecture, operates via iterative weight optimization using the error backpropagation principle, thereby achieving superior convergence and predictive accuracy [17]. Unlike conventional regression models, BPANN does not rely on assumptions of data distribution or variable independence and is capable of capturing subtle nonlinear relationships between dose parameters and toxicity [10]. In the present study, the BPANN-based model achieved excellent discrimination, with areas under the ROC curve for moderate/severe xerostomia reaching 0.97 at 1 year and 0.89 at 2 years post-radiotherapy, demonstrating feasibility and robustness in the modern IMRT/tomotherapy era.

Through integrated correlation analysis and model interpretation, three predictors—age, gender, and SMG Dmean—emerged as the strongest determinants of xerostomia risk. Older age was associated with persistently higher xerostomia probability, likely due to diminished regenerative capacity of acinar cells, reduced microvascular perfusion, and baseline glandular atrophy [18]. Female patients exhibited higher susceptibility than males, potentially explained by hormonal influences and increased prevalence of post-radiotherapy insomnia or mucosal dryness [19]. These findings are consistent with prior studies reporting gender-related variability in salivary gland radiosensitivity and recovery [20].

Although PGs and OC are traditionally emphasized in planning, our results reinforce the key role of the SMGs in maintaining baseline saliva production, especially during rest. Damage to SMGs markedly reduces unstimulated saliva output and accelerates oral dryness, even with adequate PG sparing [11]. Interestingly, while we incorporated TGs into the BPANN model (the first attempt in this domain), TG dose did not significantly correlate with xerostomia severity. Potential reasons include the predominance of nasopharyngeal carcinoma in our cohort (hence lower TG doses due to beam geometry) and compensatory function from minor glands. Nonetheless, the inclusion of TG parameters enriches the conceptual framework of comprehensive salivary gland sparing.

Beyond technical performance, our study underlines the clinical applicability of BPANN modelling. By linking patient-reported XQ with dosimetric metrics, the model bridges objective radiation parameters and subjective symptom burden, offering a personalized prediction framework. In future work, this framework could be integrated into adaptive radiotherapy workflows, enabling real-time toxicity risk estimation and individualized replanning to optimize both tumor control and normal tissue preservation.

However, several limitations deserve acknowledgement. First, the study was conducted at a single center with a moderate sample size; external validation using multi-institutional datasets is required, as the current absence of independent multicenter patient cohorts restricts the generalizability of our BPANN model’s clinical predictive performance across diverse radiotherapy centers and patient populations. Second, BPANN models can be prone to local minima and overfitting, especially with relatively small, homogeneous datasets. Third, our model included only clinical and dosimetric variables; emerging biomarkers (e.g., radiomics, salivary metabolomics, genetic markers) may further enhance predictive performance. Fourth, the current BPANN algorithm lacks a simplified, user-friendly clinical interface, such as a visualized nomogram or accessible online risk calculator, which hinders its direct, real-time application during routine radiotherapy planning. Correspondingly, the original conclusion stating that BPANN is a “recommended” clinical predictive tool was overstated, as robust external cohort validation and translational interface development are mandatory prerequisites before formal clinical recommendation, consistent with standard machine learning reporting norms for radiation toxicity prediction outlined in landmark IJROBP machine learning guidance. Our preliminary internal validation only supports the methodological superiority of this TG-integrated neural network within our single-center dataset, rather than confirming its ready-for-clinic utility. Lastly, comparative performance between BPANN and more advanced deep-learning or ensemble methods remains unexplored.

For future translational research, we plan two core follow-up works to resolve the above translational barriers: we will collaborate with multiple oncology centers to recruit a large independent external cohort receiving identical comprehensive salivary gland-sparing HT for formal external validation of the BPANN model; simultaneously, we will convert the trained BPANN algorithm into a simplified visual auxiliary prediction tool (nomogram-style risk calculator) compatible with mainstream radiotherapy planning systems, to improve clinical operability and facilitate routine bedside risk assessment. Subsequent prospective multi-center trials will further verify the clinical value of this predictive framework for individualized gland-sparing radiotherapy design.

Overall, our findings confirm that BPANN constitutes a powerful, and feasible tool for predicting radiation-induced xerostomia at 1 and 2 years post-therapy. By integrating clinical and dosimetric factors, the model achieves robust predictive accuracy, reinforcing the clinical value of SMG sparing, particularly among older and female patients.

### Conclusions

In conclusion, multivariate analysis revealed that age, gender, and SMG radiation dose are critical determinants of radiation-induced xerostomia. The BPANN prediction model demonstrated high accuracy and provides a practical framework for individualized risk assessment in head and neck radiotherapy. Incorporation of machine-learning-based models into adaptive planning may facilitate personalized gland-sparing strategies, optimize tumor control, and improve post-treatment quality of life.

## Supplementary Material

Suppl 1 Predictive indicators of the BPANN model at 1 year post-radiotherapy.

Suppl 2 Predictive indicators of the BPANN Model at 2 years post-radiotherapy.

## Data Availability

The data and materials supporting this study are included in the article and supplementary files. Further inquiries may be directed to the corresponding author.
