# Supplementary material for: Radiation-Induced Xerostomia in Patients With Head and Neck Cancer Treated With Comprehensive Salivary Gland-Sparing Helical Tomotherapy Technique Based on a Back Propagation Artificial Neural Network
Source: J Clin Med Res. 2026 Sep 26;18(9):623–31. doi: 10.14740/jocmr6633 (PMC13661088; doi:10.14740/jocmr6633)
Supplement: Supplementary file 1 [file jocmr-18-09-623-s001.docx]

**Suppl 1.** Predictive Indicators of the BPANN Model at 1 Year Post-Radiotherapy

| Items | No xerostomia | Mild xerostomia | Moderate/Severe  xerostomia |
| --- | --- | --- | --- |
| Sensitivity | 0.6923 | 0.8824 | 0.6250 |
| Specificity | 0.9400 | 0.7586 | 0.9149 |
| Positive prediction value | 0.7500 | 0.8108 | 0.7143 |
| Negative prediction value | 0.9216 | 0.8462 | 0.8776 |
| Prevalence | 0.2063 | 0.5397 | 0.2540 |
| Detection | 0.1429 | 0.4762 | 0.1587 |
| Detection prevalence | 0.1905 | 0.5873 | 0.2222 |
| Balanced accuracy | 0.8162 | 0.8205 | 0.7699 |
| Brier scores | 0.112 | 0.115 | 0.119 |
| Accuracy（95%CI） | 0.7778 (0.6554 - 0.8728) | | |
| Kappa | 0.6216 | | |
| Mcnemar's test p-value | 0.6870 | | |
