# Supplementary material for: Radiation-Induced Xerostomia in Patients With Head and Neck Cancer Treated With Comprehensive Salivary Gland-Sparing Helical Tomotherapy Technique Based on a Back Propagation Artificial Neural Network
Source: J Clin Med Res. 2026 Sep 26;18(9):623–31. doi: 10.14740/jocmr6633 (PMC13661088; doi:10.14740/jocmr6633)
Supplement: Supplementary file 2 [file jocmr-18-09-623-s002.docx]

**Suppl 2.** Predictive Indicators of the BPANN Model at 2 Years Post-Radiotherapy

| Items | No xerostomia | Mild xerostomia | Moderate/Severe  xerostomia |
| --- | --- | --- | --- |
| Sensitivity | 1.0000 | 0.9091 | 0.5714 |
| Specificity | 0.8333 | 0.9808 | 1.0000 |
| Positive prediction value | 0.9375 | 0.9091 | 1.0000 |
| Negative prediction value | 1.0000 | 0.9808 | 0.9492 |
| Prevalence | 0.7143 | 0.1746 | 0.1111 |
| Detection rate | 0.7143 | 0.1587 | 0.0635 |
| Detection prevalence | 0.7619 | 0.1746 | 0.0635 |
| Balanced accuracy | 0.9167 | 0.9449 | 0.7857 |
| Brier scores | 0.054 | 0.072 | 0.069 |
| Accuracy（95%CI） | 0.9365 (0.8453 - 0.9824) | | |
| Kappa | 0.8482 | | |
| Mcnemar's test p-value | 0.2615 | | |
